# Supplementary material for: Mutational landscape of triple-negative breast cancer in African American women
Source: Nat Genet. 2025 Aug 26;57(9):2166–76. doi: 10.1038/s41588-025-02322-y (PMC12425817; doi:10.1038/s41588-025-02322-y)
Supplement: Supplementary file 1 — Supplementary Note and Supplementary Figs. 1–12. [file 41588_2025_2322_MOESM1_ESM.pdf]

nature genetics

Supplementary information https://doi.org/10.1038/s41588-025-02322-y

Mutational landscape of triple-negative breast cancer in African American women

In the format provided by the authors and unedited

Supplementary Information

Table of Contents

Supplementary Note 2

Supplementary Fig.1. Somatic mutation catalog of triple-negative breast cancer from 462 African American women 5

Supplementary Fig.2. Tumor mutation burden of triple-negative breast cancer from African American women 6

Supplementary Fig.3. The differences in somatic and germline mutations and mutational signatures by TNBC transcriptional subtype 7

Supplementary Fig.4. Significant copy number aberrations of triple-negative breast cancer from African American women 8

Supplementary Fig.5. COSMIC mutational signatures extracted from triple-negative breast cancer from African American women 9

Supplementary Fig.6. Correlations of mutational signatures with demographic and clinical variables 10

Supplementary Fig.7. Gene ontology enrichment analysis of mutational signature-based Subtype 3 (high aging and low HRD) vs. Subtype 1 (low aging and high HRD) 11

Supplementary Fig.8. Principal component analysis of genetic ancestry in African American women with triple-negative breast cancer 12

Supplementary Fig.9. Associations of African ancestry with somatic mutations in triple-negative breast cancer from African American women 13

Supplementary Fig.10. Predicted number of neoantigens and number of non-synonymous missense mutations 14

Supplementary Fig.11. Fusion plots for recurrent fusion mutations and those of potential biological significance 15

Supplementary Fig.12. Association of CD274 (PD-L1) mRNA expression and copy number aberrations 16

References 17

Supplementary Note

Patient populations

B-CAUSE (Breast Cancer in African Americans: Understanding Somatic Mutations and Etiology) Study pooled data and biospecimens from the following five population-based breast cancer studies with large number of African American women in the US to investigate epidemiological and clinical significance of tumor somatic mutations. B-CAUSE Study was supported in part by National Institutes of Health grant nos. R01 CA228156.

The Women's Circle of Health Study (WCHS) is a case-control study initiated in 2002 to examine risk factors for aggressive breast cancer in African American and White women[1,2]. Cases were first identified from hospitals in metropolitan New York City and subsequently through New Jersey State Cancer Registry using rapid case ascertainment. Upon consent, patients complete an in-depth interview on known and suspected risk factors for breast cancer. Follow-up for mortality outcomes was conducted by data linkage with the New Jersey State Cancer Registry as part of the Women's Circle of Health Follow-up Study (WCHSFS)[3]. WCHS is supported in part by National Institutes of Health grant nos. R01 CA100598 and WCHSFS by R01 CA185623.

The Black Women's Health Study (BWHS) is a U.S.-based prospective cohort study that began in 1995 when 59,000 self-identified African American women 21-69 years of age completed a baseline health questionnaire[4]. Updated information on breast cancer risk factors and self-report of new breast cancers are obtained via biennial follow-up questionnaires. Incident breast cancers are also ascertained through linkage to 24 state cancer registries that, together, cover the state of residence for >95% of participants. Medical record and cancer registry data are sought for all participants who report a diagnosis of breast cancer. BWHS is supported in part by National Institutes of Health grant nos. UM1 CA164974.

The Southern Community Cohort Study (SCCS) was initiated in 2002 to study health disparities and enrolled approximately 86,000 adults in 12 southeastern states[5]. Nearly 70% of participants are African American. Extensive epidemiological data at baseline were collected. Incident breast cancer cases are identified via linkage to state cancer registries, and clinical data from the cancer registries and supplemented by pathology reports and medical records. SCCS is supported in part by National Institutes of Health grant nos. R01 CA092447 and U01 CA202979.

The Nashville Breast Health Study (NBHS) is a population-based case-control study of incident breast cancer among women in the Nashville area initiated in 2004 and later expanded to the entire state to increase the sample size for African American women[6]. Eligible cases were identified primarily through the Tennessee State Cancer Registry. All participants were interviewed to obtain information related to risk factors for breast

cancer. NBHS is supported in part by National Institutes of Health grant nos. R01 CA100374.

The Southern Tri-State Breast Health Study (STSBHS) was launched in 2013 to recruit African American breast cancer patients in Tennessee, Georgia, and South Carolina using the established protocols and study instruments developed in the NBHS. STSBHS is supported as part of the NCI-funded Meharry-Vanderbilt partnership program (U54 CA91405).

As a part of the routine workflow of all studies, blood, saliva, and/or mouthwash samples were collected as the source of genomic DNA, and pathology reports were requested and received along with formalin-fixed paraffin-embedded (FFPE) tumor tissues. Tumor Immunohistochemical (IHC) data from the pathology report are used to classify breast cancer subtypes[7]. For this project in B-CAUSE Study, we included 513 patients self-identified as African American women who were diagnosed with TNBC and had banked tumor tissues and genomic DNA available for sequencing.

Publicly available TNBC mutation datasets

For comparison to somatic mutations in TNBC patients from African American women in B-CAUSE, publicly available data were aggregated from Asian women in Fudan University Shanghai Cancer Center (FUSCC) and NHW women in The Cancer Genome Atlas (TCGA), the Sweden Cancerome Analysis Network – Breast (SCAN-B), and the Molecular Taxonomy of Breast Cancer International Consortium (METABRIC).

TCGA was a large cancer genomic project established by the US National Institutes of Health (NIH) to chart the genomic landscape of multiple adult cancers using multi-omics platform, with public data release and sharing. For breast cancer, genomic data were publicly available from 1,257 samples[8]. Somatic mutation data from whole-exome sequencing (version 2016-12-29) were downloaded from UCSC Xena, which included 69 patients identified as NHW with TNBC.

SCAN-B is a multicenter study in the South of Sweden to analyze breast cancers with next-generation genomic technologies for translational research in a population-based manner and integrated with healthcare. In the 2019 Nature Medicine publication, whole genome sequencing was performed for 254 TNBC, and the mutation data were downloaded from the URL provide in the publication[9].

METABRIC was a two-center study at Cambridge Research Institute in the UK and the British Columbia Cancer Centre Canada with targeted sequencing data of 173 genes from 1,980 primary breast cancer samples, including 320 patients who could be identified as TNBC based on clinical annotation data[10]. Clinical and genomic data were downloaded from cBioPortal[11].

FUSCC was a single-center study of 504 consecutive Chinese patients diagnosed with TNBC and treated at the Fudan University Shanghai Cancer Center (FUSCC). Whole-exome sequencing was performed, and data were available from 279 patients downloaded from the URL provided in the publication[12].

Supplementary Fig.1. Somatic mutation catalog of triple-negative breast cancer from 462 African American women

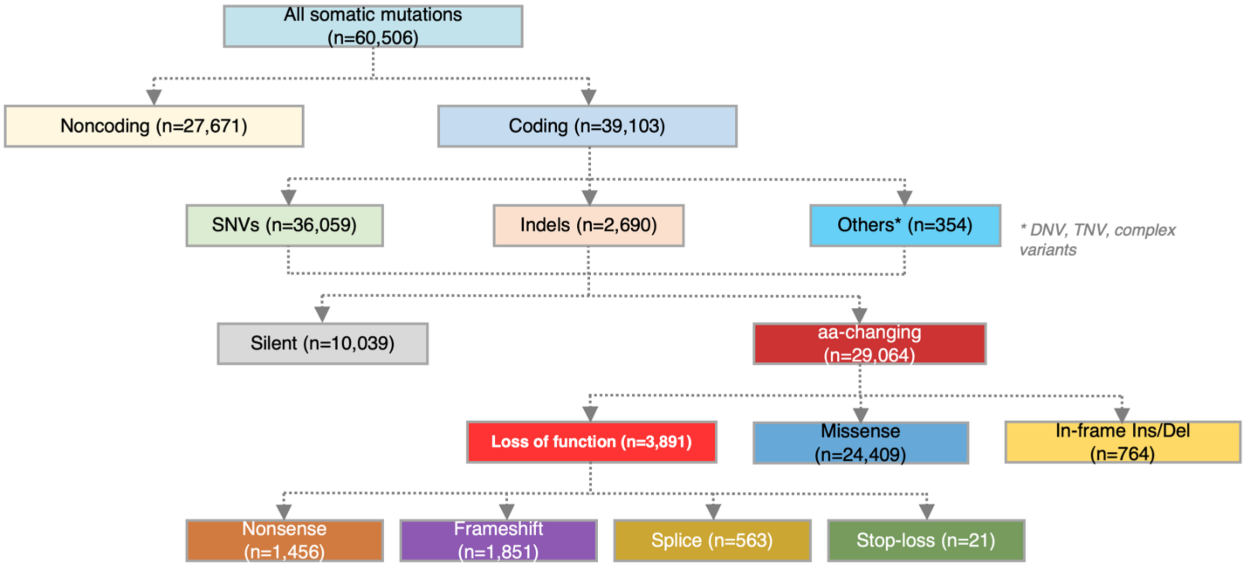

Supplementary Fig.2. Tumor mutation burden of triple-negative breast cancer from African American women

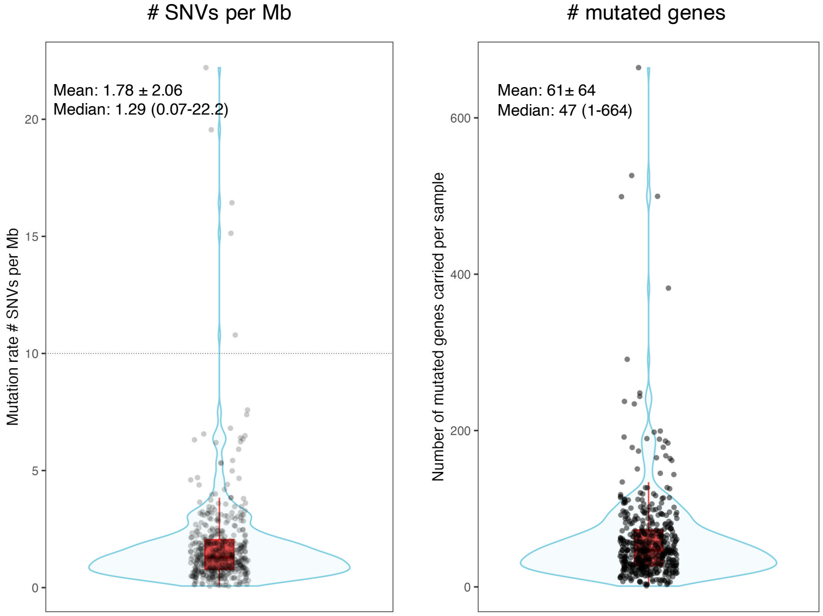

*5 hypermutated tumors with >10 SNVs / Mb

Supplementary Fig.3. The differences in somatic and germline mutations and mutational signatures by TNBC transcriptional subtype

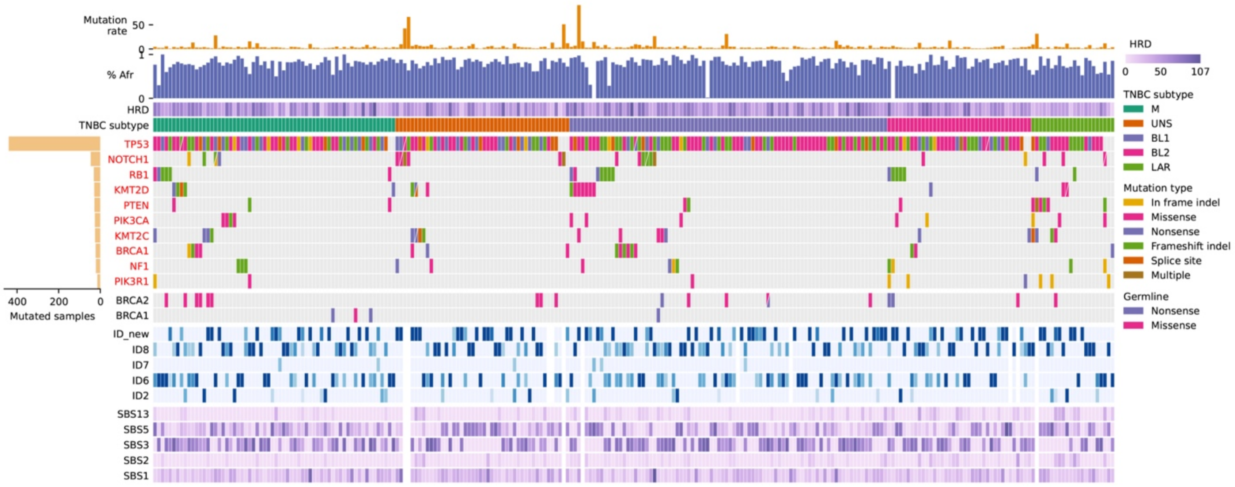

Supplementary Fig.4. Significant copy number aberrations of triple-negative breast cancer from African American women

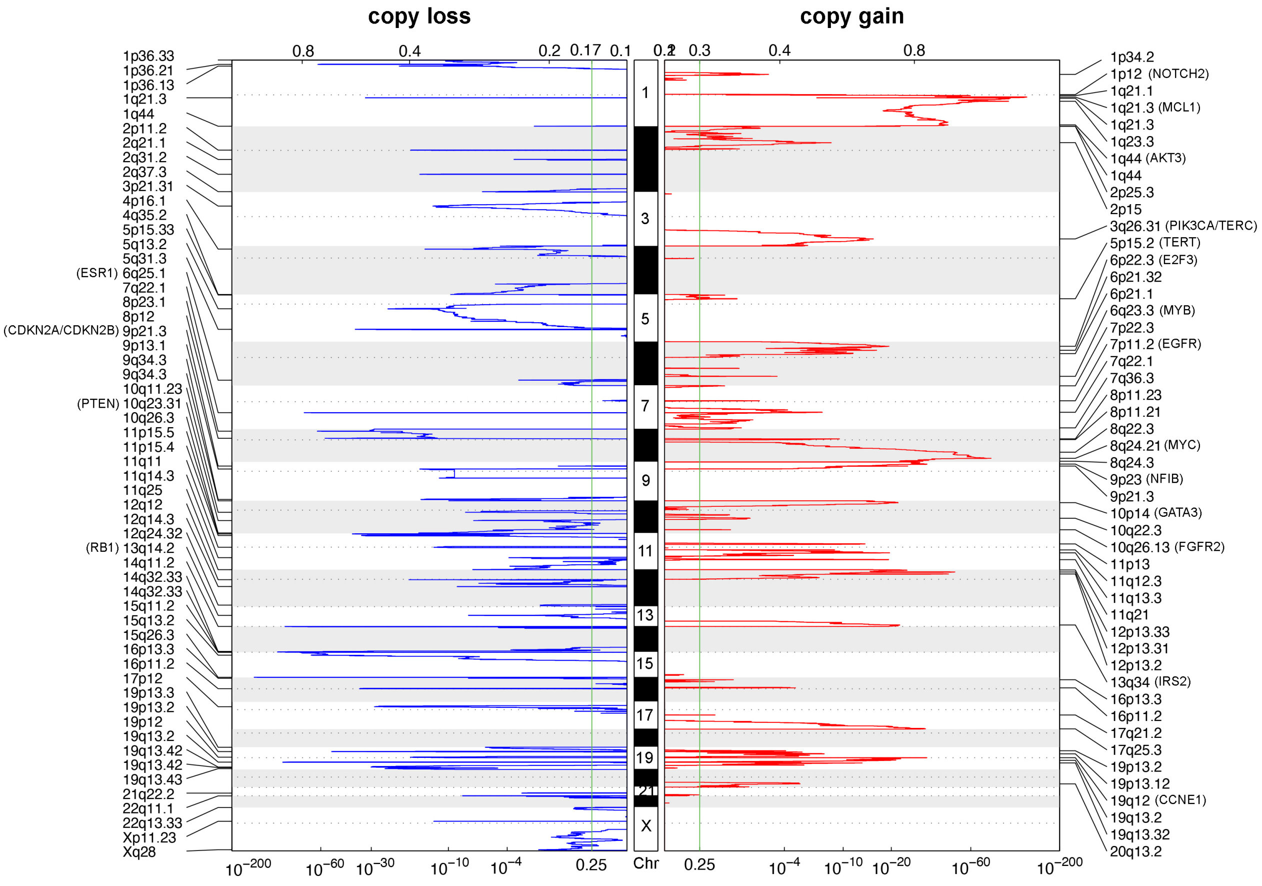

Supplementary Fig.5. COSMIC mutational signatures extracted from triple-negative breast cancer from African American women

▪ Single-base substitutions signatures

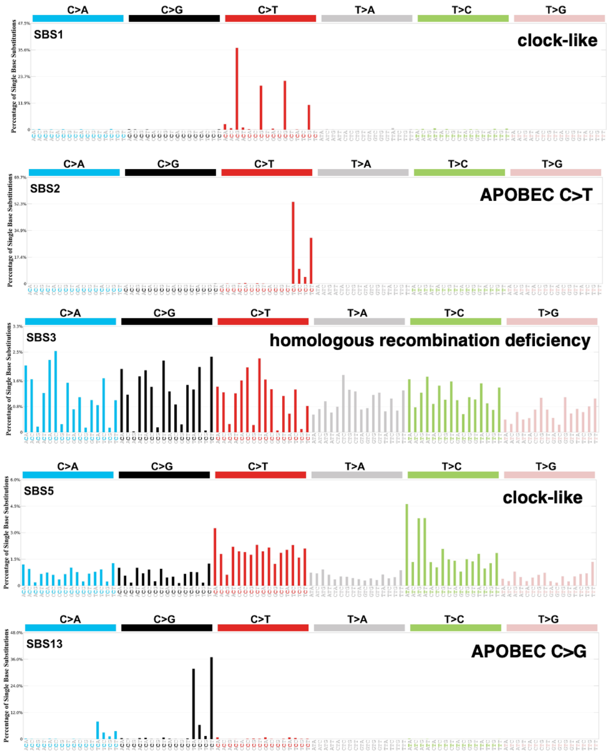

▪ Insertion/deletion signatures

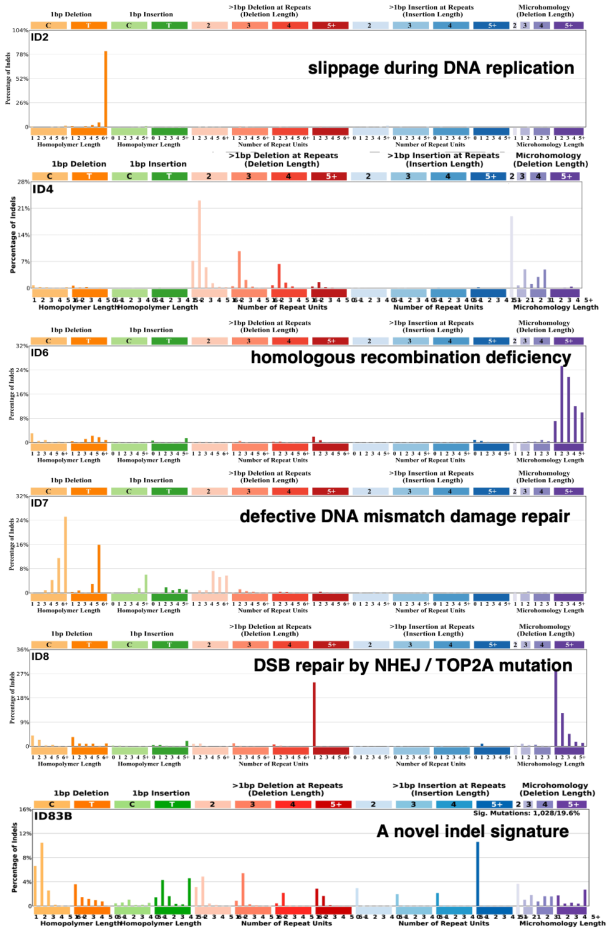

Supplementary Fig.6. Correlations of mutational signatures with demographic and clinical variables

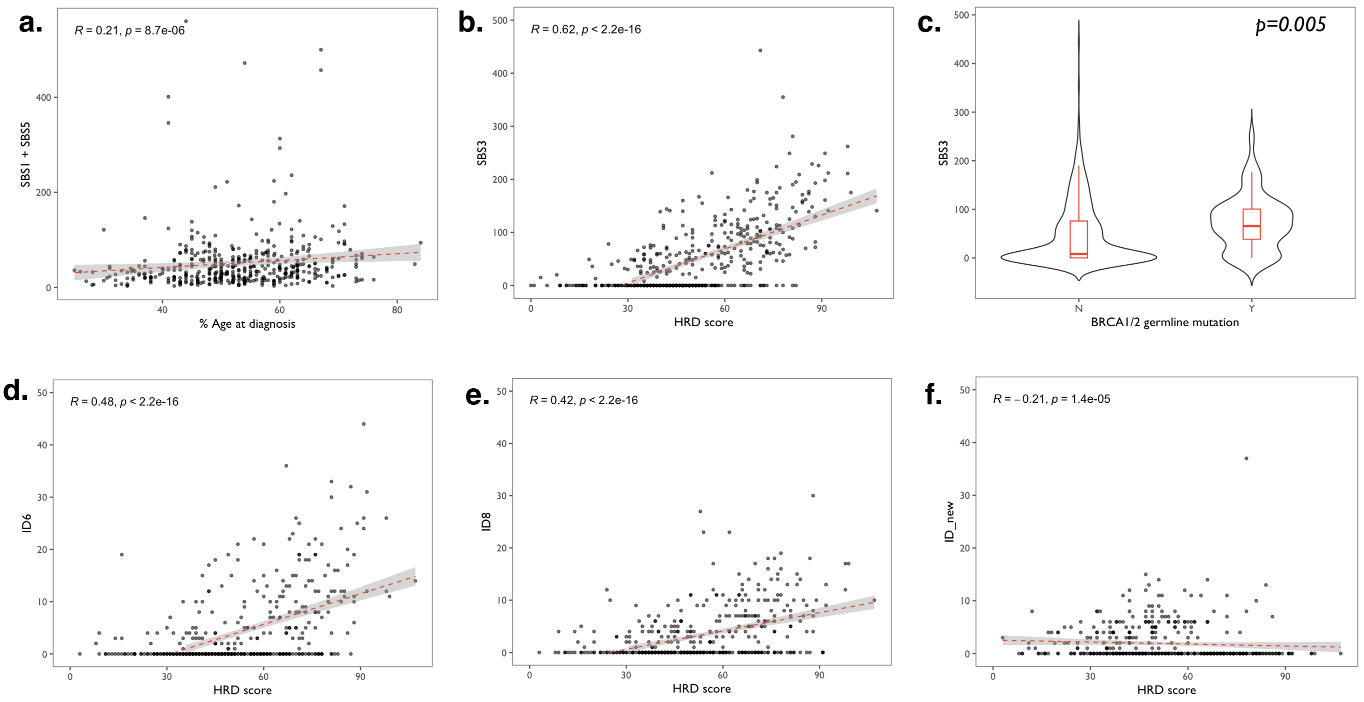

Supplementary Fig.7. Gene ontology enrichment analysis of mutational signature-based Subtype 3 (high aging and low HRD) vs. Subtype 1 (low aging and high HRD)

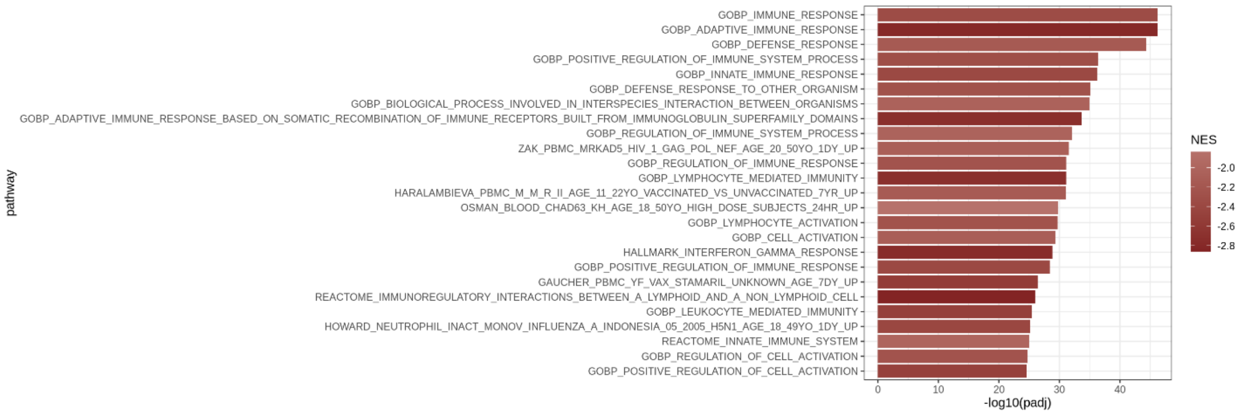

Supplementary Fig.8. Principal component analysis of genetic ancestry in African American women with triple-negative breast cancer

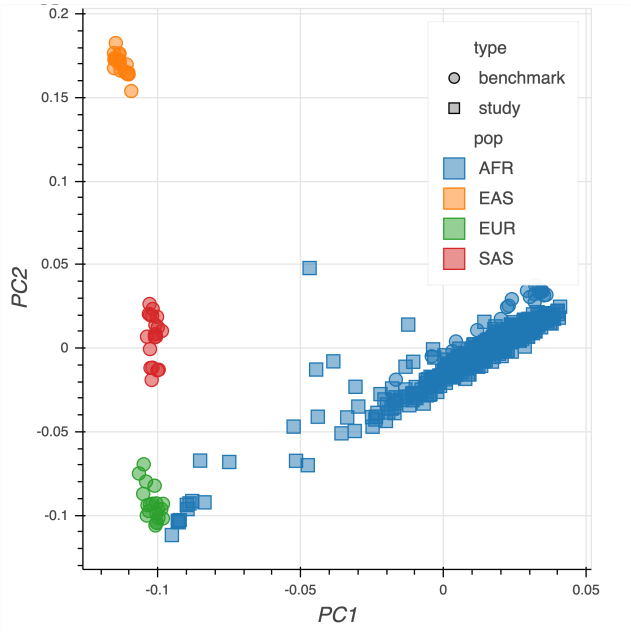

Supplementary Fig.9. Associations of African ancestry with somatic mutations in triple-negative breast cancer from African American women

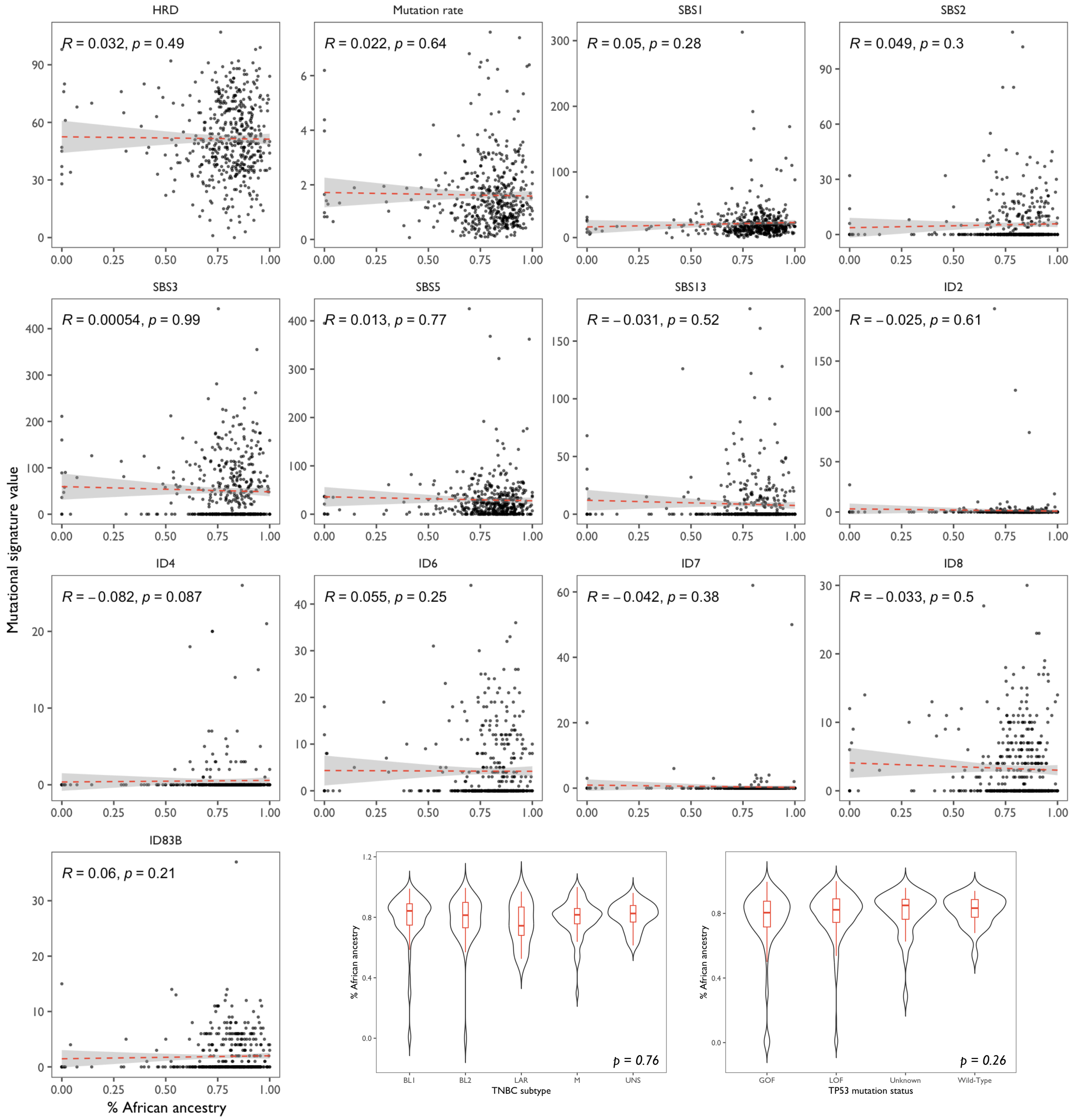

Supplementary Fig.10. Predicted number of neoantigens and number of non-synonymous missense mutations

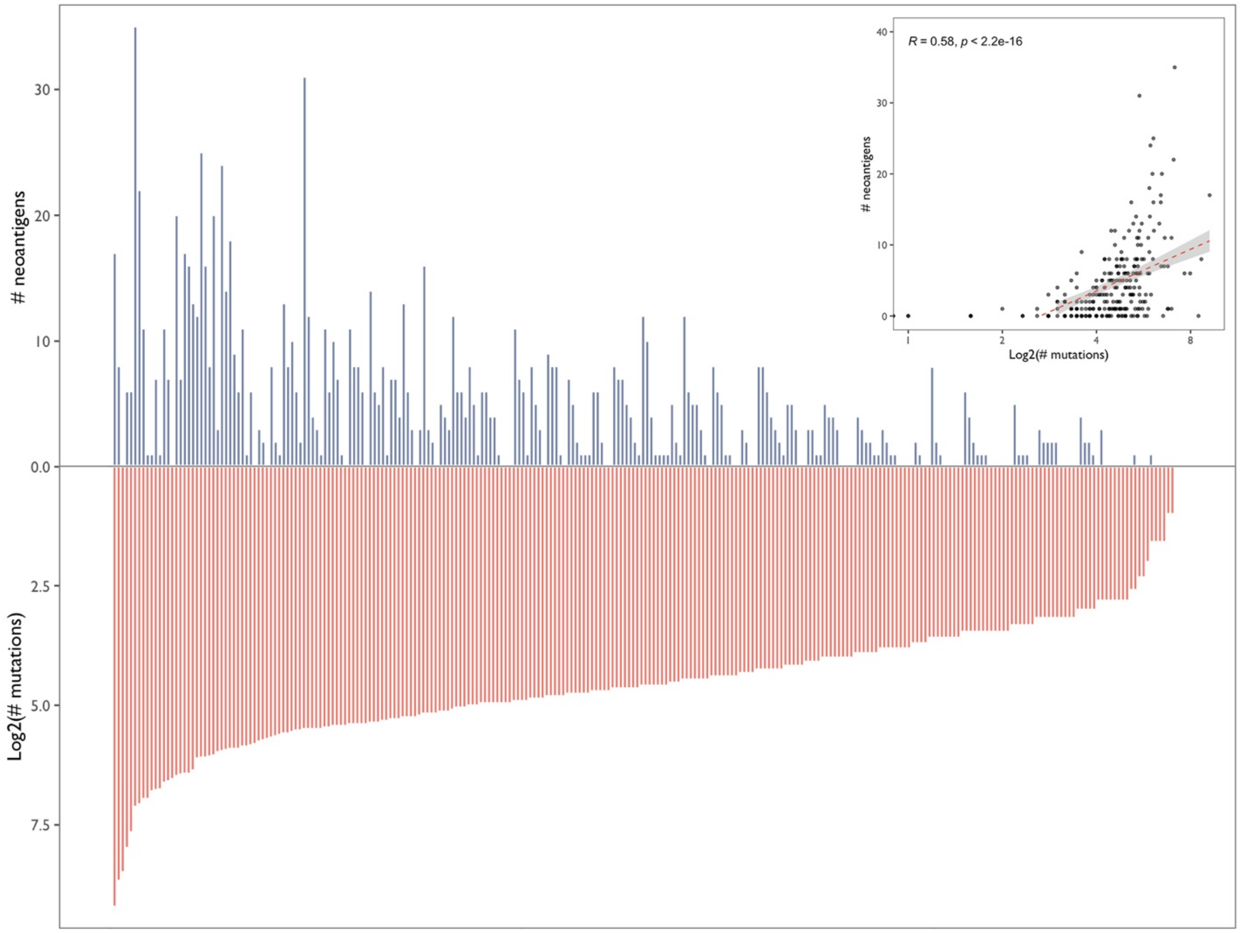

Supplementary Fig.11. Fusion plots for recurrent fusion mutations and those of potential biological significance

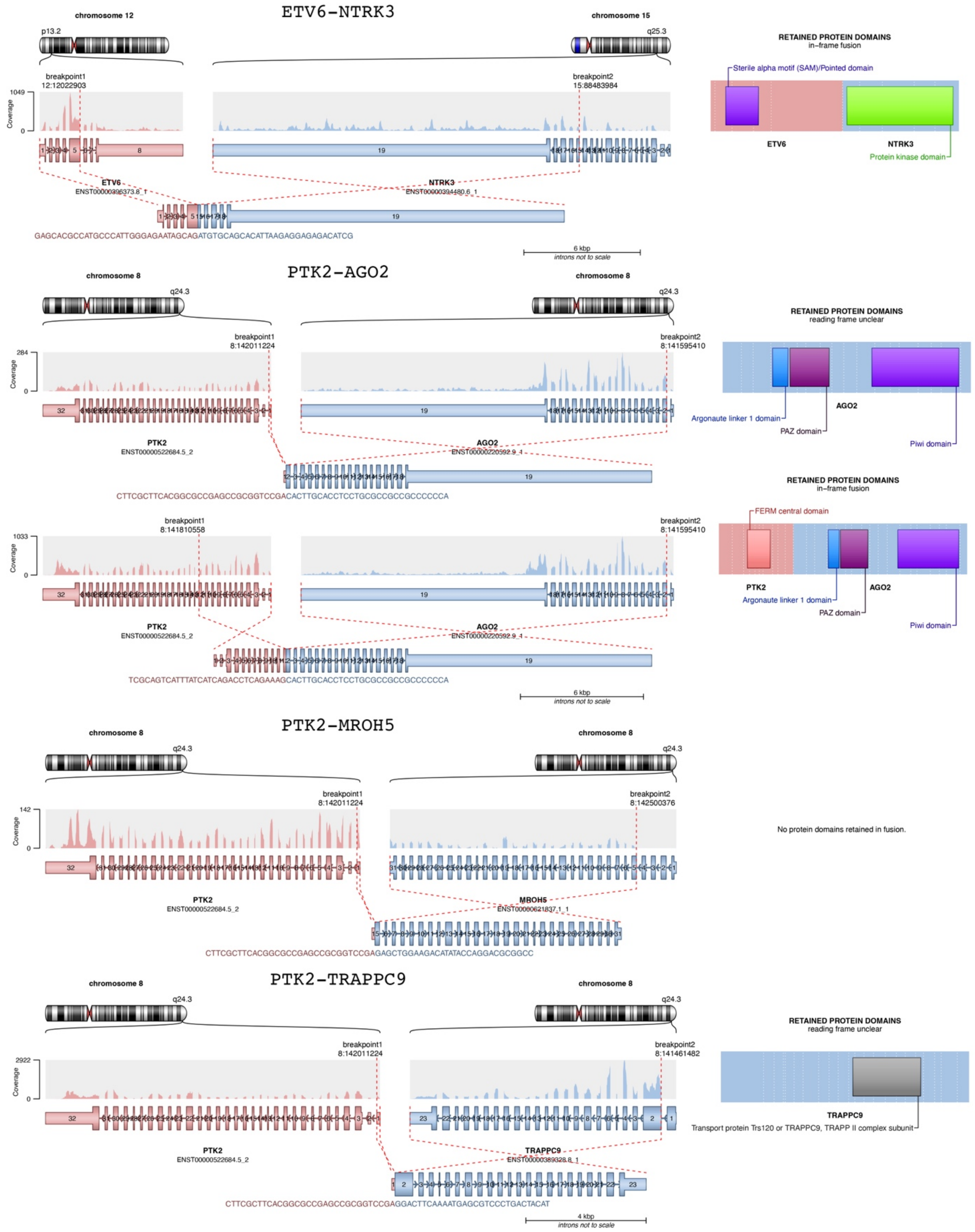

Supplementary Fig.12. Association of CD274 (PD-L1) mRNA expression and copy number aberrations

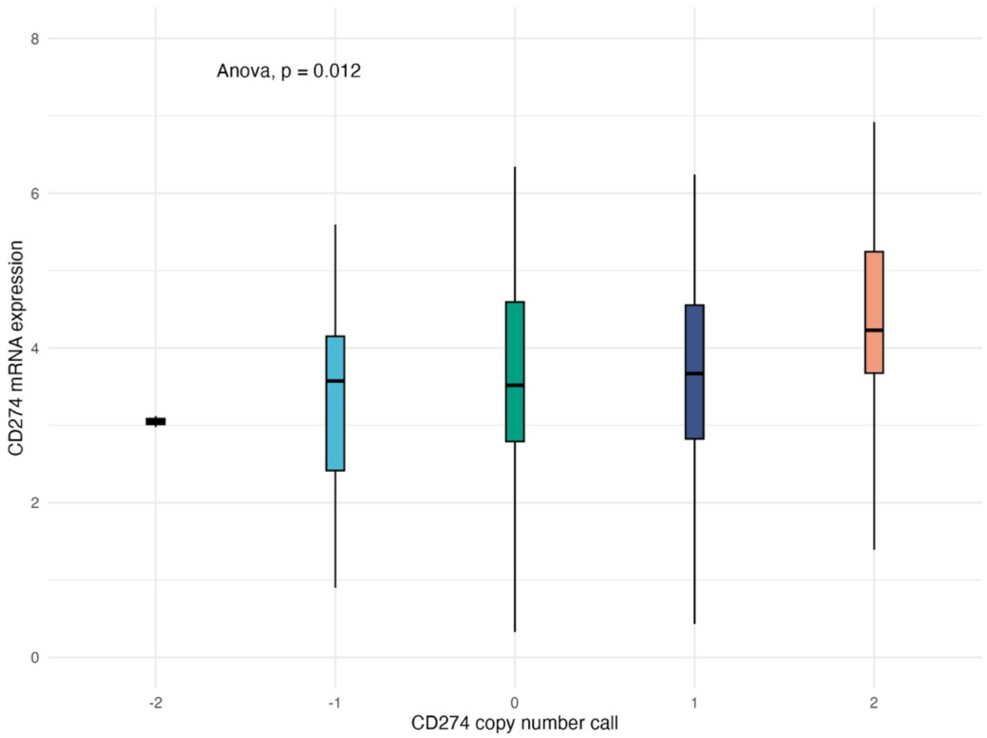

References

1 Ambrosone, C. B. *et al.* Conducting Molecular Epidemiological Research in the Age of HIPAA: A Multi-Institutional Case-Control Study of Breast Cancer in African-American and European-American Women. *J Oncol* **2009**, 871250 (2009).

2 Bandera, E. V. *et al.* Rethinking sources of representative controls for the conduct of case-control studies in minority populations. *BMC medical research methodology* **13**, 71 (2013).

3 Bandera, E. V. *et al.* The Women's Circle of Health Follow-Up Study: a population-based longitudinal study of Black breast cancer survivors in New Jersey. *J Cancer Surviv* **14**, 331-346 (2020).

4 Palmer, J. R., Rao, R. S., Adams-Campbell, L. L. & Rosenberg, L. Height and breast cancer risk: results from the Black Women's Health Study (United States). *Cancer causes & control : CCC* **12**, 343-348 (2001).

5 Signorello, L. B. *et al.* Southern community cohort study: establishing a cohort to investigate health disparities. *J Natl Med Assoc* **97**, 972-979 (2005).

6 Zheng, W. *et al.* Genome-wide association study identifies a new breast cancer susceptibility locus at 6q25.1. *Nature genetics* **41**, 324-328 (2009).

7 Allott, E. H. *et al.* Performance of Three-Biomarker Immunohistochemistry for Intrinsic Breast Cancer Subtyping in the AMBER Consortium. *Cancer epidemiology, biomarkers & prevention : a publication of the American Association for Cancer Research, cosponsored by the American Society of Preventive Oncology* **25**, 470-478 (2016).

8 Cancer Genome Atlas, N. Comprehensive molecular portraits of human breast tumours. *Nature* **490**, 61-70 (2012).

9 Staaf, J. *et al.* Whole-genome sequencing of triple-negative breast cancers in a population-based clinical study. *Nature medicine* **25**, 1526-1533 (2019).

10 Pereira, B. *et al.* The somatic mutation profiles of 2,433 breast cancers refines their genomic and transcriptomic landscapes. *Nat Commun* **7**, 11479 (2016).

11 Cerami, E. *et al.* The cBio cancer genomics portal: an open platform for exploring multidimensional cancer genomics data. *Cancer Discov* **2**, 401-404 (2012).

12 Jiang, Y. Z. *et al.* Genomic and Transcriptomic Landscape of Triple-Negative Breast Cancers: Subtypes and Treatment Strategies. *Cancer Cell* **35**, 428-440 e425 (2019).
