# Supplementary material for: Mutational landscape of triple-negative breast cancer in African American women
Source: Nat Genet. 2025 Aug 26;57(9):2166–76. doi: 10.1038/s41588-025-02322-y (PMC12425817; doi:10.1038/s41588-025-02322-y)
Supplement: Supplementary file 2 — Reporting Summary [file 41588_2025_2322_MOESM2_ESM.pdf]

nature portfolio

Corresponding author(s): Song Yao, MD, PhD

Last updated by author(s): Jun 8, 2025

# Reporting Summary

Nature Portfolio wishes to improve the reproducibility of the work that we publish. This form provides structure for consistency and transparency in reporting. For further information on Nature Portfolio policies, see our Editorial Policies and the Editorial Policy Checklist.

## Statistics

For all statistical analyses, confirm that the following items are present in the figure legend, table legend, main text, or Methods section.

| n/a | Confirmed | |
|---|---|---|
| ☐ | ☒ | The exact sample size (*n*) for each experimental group/condition, given as a discrete number and unit of measurement |
| ☐ | ☒ | A statement on whether measurements were taken from distinct samples or whether the same sample was measured repeatedly |
| ☐ | ☒ | The statistical test(s) used AND whether they are one- or two-sided<br>*Only common tests should be described solely by name; describe more complex techniques in the Methods section.* |
| ☐ | ☒ | A description of all covariates tested |
| ☐ | ☒ | A description of any assumptions or corrections, such as tests of normality and adjustment for multiple comparisons |
| ☐ | ☒ | A full description of the statistical parameters including central tendency (e.g. means) or other basic estimates (e.g. regression coefficient) AND variation (e.g. standard deviation) or associated estimates of uncertainty (e.g. confidence intervals) |
| ☐ | ☒ | For null hypothesis testing, the test statistic (e.g. *F*, *t*, *r*) with confidence intervals, effect sizes, degrees of freedom and *P* value noted<br>*Give P values as exact values whenever suitable.* |
| ☒ | ☐ | For Bayesian analysis, information on the choice of priors and Markov chain Monte Carlo settings |
| ☒ | ☐ | For hierarchical and complex designs, identification of the appropriate level for tests and full reporting of outcomes |
| ☐ | ☒ | Estimates of effect sizes (e.g. Cohen's *d*, Pearson's *r*), indicating how they were calculated |

*Our web collection on statistics for biologists contains articles on many of the points above.*

## Software and code

Policy information about availability of computer code

| | |
|---|---|
| Data collection | No specific code or software was used as a central part of the data collection for this study. |
| Data analysis | No custom code or special software/program was used that was central to the data analyses. The following standard open-source software and programs were used in the data analysis:<br><br>RcwlPipelines R package (v.1.20.0)<br>BWA-MEM aligner (v.0.7.18)<br>Strelka2 (v.2.9.10)<br>MuSE (v.2.0)<br>VarDict (v.1.1)<br>Mutect2 (v.4.1.0.0)<br>FACETS (v.0.6.2)<br>GISTIC2 (v.2.0.23)<br>MutSigCV (v.1.3.01)<br>MutSig2CV<br>MuSiC (v.0.4)<br>sigProfilerExtractor (v.1.1.24)<br>ALStructure R package<br>FastQC (v.0.12.1)<br>Cutadapt (v.2.0)<br>STAR-Aligner (v.2.7.11b) |

RSeQC (v.5.0.1)
mRIN (v.1.2.0)
RSEM (v.1.3.3)
sva-ComBat-seq R package (v.3.52.0)
Arriba (v.2.4.0)
STAR-fusion (v.1.13.0)
scarHRD R package (v.0.1.1)
GSEA (v.4.3.3)
MSigDB (v.2023.2)
OncoKB API (v.3.4.1)
R programming (v.4.4.0)

For manuscripts utilizing custom algorithms or software that are central to the research but not yet described in published literature, software must be made available to editors and reviewers. We strongly encourage code deposition in a community repository (e.g. GitHub). See the Nature Portfolio guidelines for submitting code & software for further information.

# Data

Policy information about availability of data

All manuscripts must include a data availability statement. This statement should provide the following information, where applicable:

- Accession codes, unique identifiers, or web links for publicly available datasets
- A description of any restrictions on data availability
- For clinical datasets or third party data, please ensure that the statement adheres to our policy

The Cancer Genome Atlas (TCGA) data: https://xena.ucsc.edu/public/. The Sweden Cancerome Analysis Network – Breast (SCAN-B): https://data.mendeley.com/datasets/2mn4ctdpxp/3. The Molecular Taxonomy of Breast Cancer International Consortium (METABRIC): https://github.com/cBioPortal/datahub/tree/master/reference_data/gene_panels. The Fudan University Shanghai Cancer Center (FUSCC): https://figshare.com/articles/dataset/A_comprehensive_genomic_and_transcriptomic_dataset_of_triple-negative_breast_cancers/19783498/5. Human Reference Genome (GRCh37): https://www.ncbi.nlm.nih.gov/datasets/genome/GCF_000001405.13/
The whole-exome sequencing data of matched tumor and normal TNBC samples in B-CAUSE has been deposited to the database of Genotypes and Phenotypes (dbGaP), with access number phs003962.v1.p1.

# Human research participants

Policy information about studies involving human research participants and Sex and Gender in Research.

**Reporting on sex and gender**

All patients were self-identified as African American females, because this study focuses specifically on breast cancer in African American females due to their disproportionately high disease burden. Breast cancer in biological males is rare.

**Population characteristics**

The patient descriptive characteristics are summarized in Extended Data Table 1. All patients were self-identified as Black females. The average (±sd) age at diagnosis was 53 (±11) years, with 38% before age 50. Twenty-two percent (22%) reported first-degree family history of breast cancer and 4% reported family history of ovarian cancer. Most cases were diagnosed at stage I (40%) or II (44%) and poorly differentiated (90%).

**Recruitment**

This study did not conduct any new patient recruitment work, but pooled existing data and biospecimens from the following five population-based breast cancer studies with large number of African American (AA) females in the US to investigate epidemiological and clinical significance of tumor somatic mutations.

The Women's Circle of Health Study (WCHS) is a case-control study initiated in 2002 to examine risk factors for aggressive breast cancer in AA and White females. Cases were first identified from hospitals in metropolitan New York City and subsequently through New Jersey State Cancer Registry using rapid case ascertainment. Upon consent, patients complete an in-depth interview on known and suspected risk factors for breast cancer. Follow-up for mortality outcomes was conducted by data linkage with the New Jersey State Cancer Registry as part of the Women's Circle of Health Follow-up Study (WCHSFS).

The Black Women's Health Study (BWHS) is a U.S.-based prospective cohort study that began in 1995 when 59,000 self-identified AA females 21-69 years of age completed a baseline health questionnaire59. Updated information on breast cancer risk factors and self-report of new breast cancers are obtained via biennial follow-up questionnaires. Incident breast cancers are also ascertained through linkage to 24 state cancer registries that, together, cover the state of residence for >95% of participants. Medical record and cancer registry data are sought for all participants who report a diagnosis of breast cancer.

The Southern Community Cohort Study (SCCS) was initiated in 2002 to study health disparities and enrolled approximately 86,000 adults in 12 southeastern states60. Nearly 70% of participants are AA. Extensive epidemiological data at baseline were collected. Incident breast cancer cases are identified via linkage to state cancer registries, and clinical data from the cancer registries and supplemented by pathology reports and medical records.

The Nashville Breast Health Study (NBHS)61 is a population-based case-control study of incident breast cancer among females in the Nashville area initiated in 2004 and later expanded to the entire state to increase the sample size for AA females. Eligible cases were identified primarily through the Tennessee State Cancer Registry. All participants were interviewed to obtain information related to risk factors for breast cancer.

| | |
|---|---|
| | The Southern Tri-State Breast Health Study (STSBHS) was launched in 2013 to recruit AA breast cancer patients in Tennessee, Georgia, and South Carolina using the established protocols and study instruments developed in the NBHS. |
| Ethics oversight | The study protocol for human subject protection was reviewed and approved by the Institutional Review Boards of all participating institutions. The IRB protocol numbers are: Roswell Park (STUDY00000692/BDR 102718); Boston University (H-38636); Vanderbilt University Medical Center (#110190). |

Note that full information on the approval of the study protocol must also be provided in the manuscript.

# Field-specific reporting

Please select the one below that is the best fit for your research. If you are not sure, read the appropriate sections before making your selection.

☒ Life sciences ☐ Behavioural & social sciences ☐ Ecological, evolutionary & environmental sciences

For a reference copy of the document with all sections, see nature.com/documents/nr-reporting-summary-flat.pdf

# Life sciences study design

All studies must disclose on these points even when the disclosure is negative.

| | |
|---|---|
| Sample size | We assembled 512 triple-negative breast cancer with existing paired tumor and normal samples available from self-identified African American females for whole-exome sequencing from five population-based breast cancer studies in the US. These were all the eligible cases from these studies. No prior sample size calculation was performed. Of those patients, 260 patients had tumor transcriptomic data from RNA sequencing. In addition, we also included TNBC cases with tumor sequencing data from publicly available data sources, including 279 Asian cases from Fudan University Shanghai Cancer Center (FUSCC), 254 non-Hispanic White cases from the Sweden Cancerome Analysis Network – Breast (SCAN-B), 69 non-Hispanic White cases from The Cancer Genome Atlas (TCGA), and 320 non-Hispanic White cases from the Molecular Taxonomy of Breast Cancer International Consortium (METABRIC). These are the all the cases publicly available for comparison with African American cases in our data. No statistical methods were used to predetermine the sample size. |
| Data exclusions | During the data processing and QC processes for the whole-exome sequencing data, there were 20 tumor samples and one normal sample that did not reach the targeted sequencing depth, 10 samples with unmatched tumor-normal sample identity, and four samples with cryptic relatedness were removed from analysis, leaving 478 tumor-normal pairs. Tumor purity was estimated using FACETS and 16 samples with low tumor purity (<0.10) were also removed. As a result, 462 tumor-normal pairs were retained in the final analysis. |
| Replication | Two orthogonal methods were used to confirm TP53 mutations, one using transcriptomic data available from 260 cases, and the other using targeted amplicon sequencing in 338 cases where adequate tumor DNA was available after whole-exome sequencing. Both validation analyses were successful to confirm the TP53 mutations. For analyses of mutational signature-based subtypes with patient survival, meta-analysis across the three studies were performed, which showed consistent results across studies. |
| Randomization | This is an observational study to characterize the mutational landscape of TNBC in African American women, and randomization is not relevant. The analyses were adjusted for covariates to minimize possible confounding effects. |
| Blinding | Laboratory technicians who performed the sample processing and sequencing work were blinded with no information disclosed that could potentially bias their work. |

# Reporting for specific materials, systems and methods

We require information from authors about some types of materials, experimental systems and methods used in many studies. Here, indicate whether each material, system or method listed is relevant to your study. If you are not sure if a list item applies to your research, read the appropriate section before selecting a response.

## Materials & experimental systems

| n/a | Involved in the study | |
|---|---|---|
| ☒ | ☐ | Antibodies |
| ☒ | ☐ | Eukaryotic cell lines |
| ☒ | ☐ | Palaeontology and archaeology |
| ☒ | ☐ | Animals and other organisms |
| ☒ | ☐ | Clinical data |
| ☒ | ☐ | Dual use research of concern |

## Methods

| n/a | Involved in the study | |
|---|---|---|
| ☒ | ☐ | ChIP-seq |
| ☒ | ☐ | Flow cytometry |
| ☒ | ☐ | MRI-based neuroimaging |
